# The dipeptidyl peptidase-4 inhibitor Saxagliptin improves function of circulating pro-angiogenic cells from type 2 diabetic patients

**DOI:** 10.1186/1475-2840-13-92

**Published:** 2014-05-14

**Authors:** Nicol Poncina, Mattia Albiero, Lisa Menegazzo, Roberta Cappellari, Angelo Avogaro, Gian Paolo Fadini

**Affiliations:** 1 Venetian Institute of Molecular Medicine, University Hospital of Padova, Via Giustiniani, Padova 2. 35100, Italy; 2 Department of Medicine, Division of Metabolic Diseases, University Hospital of Padova, Via Giustiniani, Padova 2. 35100, Italy

**Keywords:** Regeneration, Stem cells, Angiogenesis, Cardiovascular disease

## Abstract

**Background:**

Type 2 diabetes (T2D) is associated with reduction and dysfunction of circulating pro-angiogenic cells (PACs). DPP-4 inhibitors, a class of oral agents for T2D, might possess pleiotropic vasculoprotective activities. Herein, we tested whether DPP-4 inhibition with Saxagliptin affects the function of circulating PACs from T2D and healthy subjects.

**Methods:**

PACs were isolated from T2D (n = 20) and healthy (n = 20) subjects. Gene expression, clonogenesis, proliferation, adhesion, migration and tubulisation were assessed *in vitro* by incubating PACs with or without Saxagliptin and SDF-1α. Stimulation of angiogenesis by circulating cells from T2D patients treated with Saxagliptin or other non-incretinergic drugs was assessed *in vivo* using animal models.

**Results:**

Soluble DPP-4 activity was predominant over cellular activity and was successfully inhibited by Saxagliptin. At baseline, T2D compared to healthy PACs contained less acLDL^+^Lectin^+^ cells, and showed altered expression of genes related to adhesion and cell cycle regulation. This was reflected by impaired adhesion and clonogenesis/proliferative response of T2D PACs. Saxagliptin + SDF-1α improved adhesion and tube sustaining capacity of PACs from T2D patients. CD14^+^ PACs were more responsive to Saxagliptin than CD14^-^ PACs. While Saxagliptin modestly reduced angiogenesis by mature endothelial cells, circulating PACs-progeny cells from T2D patients on Saxagliptin treatment displayed higher growth factor-inducible *in vivo* angiogenetic activity, compared to cells from T2D patients on non-incretinergic regimen.

**Conclusions:**

Saxagliptin reverses PACs dysfunction associated with T2D *in vitro* and improves inducible angiogenesis by circulating cells *in vivo*. These data add knowledge to the potential pleiotropic cardiovascular effects of DPP-4 inhibition.

## Background

Type 2 diabetes (T2D) increases the risk of cardiovascular disease 2–3 fold. This is attributed to excess endothelial damage resulting from hyperglycemia and associated metabolic abnormalities, as well as impaired vascular repair [1]. Circulating cells with vascular repair and pro-angiogenic capacity are reduced and functionally impaired in T2D [2-4], which is believed to contribute to diabetic vascular and cardiac disease [5,6]. While reduction of circulating progenitors and endothelial progenitor cells (EPCs) in diabetes is much likely caused by impaired mobilization from the bone marrow [7]. dysfunction of circulating pro-angiogenic cells (PACs) results from the adverse metabolic milieu of T2D [8]. It has been reported that EPCs/PACs reduction and dysfunction is, to some extent, reversible by drugs commonly used for the treatment of T2D [9]. Several different phenotypes and culture methods have been described for so-called “EPCs” [10], We herein focus on hematopoietic-derived early EPCs, also known as circulating angiogenic cells (CACs) or PACs, because they can be easily and reproducibly isolated from peripheral blood of healthy and diseased subjects, while late EPCs or ECFC (endothelial colony forming cells) have stochastic appearance in culture [2,10].

Dipeptidyl peptidase (DPP)-4 inhibitors (DPP-4i) are currently used for the management of T2D, as they prevent the degradation of endogenous incretin hormones (glucagon-like peptide-1 [GLP-1] and glucose-dependent insulinotropic peptide [GIP]), leading to meal-induced insulin secretion. In addition to incretins, DPP-4 has several other physiologic substrates, including cytokines, chemokines, and neurohormones that can affect vascular function and metabolism [11]. By inhibiting the enzymatic degradation of such factors, DPP-4i have the potential to exert pleiotropic cardiovascular effects [12]. Interestingly, stromal cell-derived factor (SDF)-1α is a natural DPP-4 substrate and a major regulator of stem cell mobilization [13], as well as EPCs/PACs function [14]. We have previously shown that a short-term treatment with the DPP-4i Sitagliptin is able to increase circulating EPCs in T2D, likely via SDF-1α [15]. In addition, data obtained in animal models suggest potential benefit of DPP-4 inhibition on EPC pro-angiogenic activity [16,17].

Herein, we tested the hypothesis that DPP-4i can affect the function of PACs from T2D patients, using *in vitro* and *in vivo* assays. As SDF-1α is the major candidate mediator that translates DPP-4 inhibition into improved PACs function, *in vitro* experiments were performed with and without concomitant SDF-1α supplementation.

## Methods

### Patients

The study was approved by the Ethical Committee of the University Hospital of Padova and was conducted in accordance with the principles of the Declaration of Helsinki as revised in 2008 Informed consent was obtained from patients. Type 2 diabetic patients were recruited at the outpatient clinic of the Metabolic Division, University Hospital of Padova. Healthy blood donor subjects were recruited anonymously from the local blood biobank, provided they were free from diabetes and cardiovascular disease. For diabetic patients, the following data were collected from the electronic outpatient clinic charts: age, sex, BMI, waist circumference, systolic and diastolic blood pressure. diabetes duration, HbA1c, lipid profile, concomitant risk factors, complications and medications. Coronary artery disease was defined as a past history of myocardial infarction of angina confirmed by a coronary angiography showing stenosis >70% in at least one epicardial coronary vessel, or in the presence of a non-invasive stress test indicative of inducible myocardial ischemia. Peripheral arterial disease was defined as claudication, rest pain, or ischemic diabetic foot confirmed by an angiographic or ultrasound examination. Cerebrovascular disease was defined as a past history of stroke or evidence of carotid artery stenosis >30% at a ultrasound examination. Retinopathy was defined based on digital funduscopic photography scored remotely by expert ophthalmologists. Nephropathy was defined as overt macroalbuminuria (urinary albumin/creatinin ratio [ACR] > 300 mg/g) or as chronic renal failure (estimated glomerular filtration rate [eGFR] < 60 ml/min/1.73 m^2^). As the 2.5 mg renal dose-adjusted formulation of Saxagliptin was not yet commercially available in Italy when the study was performed, none of the patients in the in vivo study had chronic renal failure. Data on medications were also collected. For the in vivo angiogenesis assay, patients on Saxagliptin treatment (n = 5) since >4 months and patients on other, non-incretinergic, regimen (n = 5) were enrolled. As treatment had been previously decided on clinical ground and was not assigned by the investigators, this did not represent a clinical trial and was not registered as such.

### Culture of pro-angiogenic cells (PACs) and functional assessment

PBMCs were isolated by density gradient centrifugation with Histopaque-1077. Cells were plated on human fibronectin-coated culture dishes and maintained in endothelial cell basal medium-2 (EBM-2). The medium was supplemented with EGM-2 MV SingleQuots containing FBS (5%), human VEGF-1, human fibroblast growth factor-2 (FGF-2), human epidermal growth factor (EGF), insulin-like growth factor-1 (IGF-1), and ascorbic acid. After 4 days in culture, nonadherent cells were removed by washing with PBS, new medium was applied, and the cells were maintained through day 7 cultured with or without Saxagliptin (Saxa, DPP-4 inhibitor) and with or without SDF-1α.

PACs obtained after 7 days of culture without stimuli were immunophenotyped by fluorescence microscopy (Leica DM 6000B) for the ability to uptake AcLDL and bind FITC-*Ulex europaeus* agglutinin Lectin. Adherent cells were first incubated with Dil-acLDL for 1 h, then counterstained with FITC-Lectin and fixed in 2% paraformaldehyde. Images were acquired with the manufacturer’s software, and assembled using Adobe Photoshop.

In separate experiments, PACs were cultured from unselected PBMCs of healthy controls in the presence of Saxagliptin and/or SDF-1α and, at the end of the 7 day culture period, CD14^+^ PACs were separated from CD14^-^ PACs using the MS Column and MiniMACS Separator (Miltenyi Biotec). Then, functional assays and gene expression were analyzed separately for CD14^+^ and CD14^-^ PACs.

#### Adhesion assay

A monolayer of human umbilical vein endothelial cells (HUVECs) was prepared 48 hours before the assay by plating 2 × 10^5^ cells (passage 5 to 8) in each well of 24-well plate. PACs were labeled with CMTMR and 1 × 10^5^ cells were added to each well and incubated for 3 hours at 37°C. Non attached cells were gently removed with PBS, and adherent PACs were fixed with 4% paraformaldehyde and counted in 10 randomly selected field.

#### Matrigel tubule assay

Matrigel (Sigma-Aldrich) was thawed and placed in 96-well plate at room temperature for 30 minutes to allow solidification. PACs (3 × 10^3^) were co-plated with 1.5 × 10^4^ human umbilical vein endothelial cells (HUVECs) and incubated at 37°C for 24 hours. The 5:1 HUVECs/PACs ration was chosen based on a preliminary dose–response experiments (not shown), which showed a suppressive effect of higher ratios on tube formation. Tubule formation was defined as a structure exhibiting a length 4 times its width. The length and the number of tubules was determined in 10 randomly selected fields.

In separate experiments performed to analyze the physical location of PACs co-cultured with HUVECs in the Matrigel tubule assay, PACs were red-labelled with the fluorescent dye Cell-Tracker Orange CMTMR (Life Technologies).

#### Migration assay

Cell migratory assays were performed using Transwell chambers with filter membranes of 3 mm pore size. Transwell chambers were inserted into the plate wells. PACs were seeded into the upper chamber (10^4^ cells per well in serum-free medium) in either the absence or the presence of SDF-1α at 37°C. At the end of the assay, 3 hours later, migrated PACs in the lower compartment were collected and counted using the flow cytometer. Results are reported as ratio of the number of migrated cells and non migrated cells.

### Flow cytometry and immunomagnetic cell sorting

For the characterization of PACs, cells were detached using EDTA and scraping. Cells were labelled with mouse anti-human PerCP-Cy5.5 CD45 (BD Pharmingen, cat# 552724), PE KDR (R&D Systems, cat# FAB357P), FITC CD68 (Dako Cytomation, cat#F7135), APC CD34 (BD Pharmingen, cat# 345804), PE CD14 (Beckman Coulter, cat# A07764), FITC CD26 (BD Pharmingen, cat# 555436), FITC CD31 (BD Pharmingen, cat# 555445). Events were acquired using a FacsCanto instrument (BD), after morphological gating in the SSC vs FSC plot. Al least 10^5^ events were acquired for each analysis.

PACs treated with and without Saxagliptin and/or SDF-1α for 7 days were trypsinized into conical tubes, washed twice with PBS and fixed in 70% ice-cold ethanol. For DNA analysis, cells were centrifuged at 200 g for 10 min at 4°C and washed twice with PBS. For cell cycle analysis, after incubation at 37°C in the dark for 15 min, DNA content of the nuclei was determined by staining nuclear DNA with propidium iodide solution (50 μg/mL, sigma, USA) containing 50 μg/mL ribonuclease A. The DNA content was measured by a flow cytometry (FacsCanto) and scored.

For immunomagnetic selection of CD14^pos^ and CD14^neg^ cells out of the initial PBMC population for PACs culture, we used the MS Column and MiniMACS Separator (Miltenyi Biotec). After isolation of PBMCs, cells were centifugated at 300 g for 10 minutes. The cell pellets were resuspended in 80 μL of buffer (MACS buffer), 20 μl of CD14 MicroBeads per 10^7^ total cells were added. After 30 minutes in the refrigerator, cells was washed by adding MACS buffer and resuspended in 500 μL of buffer. The magnetic separation was performed as described by the manufactures.

### Determination of DPP-4 activity

DPP-4 activity was determined in conditioned medium or cell extracts from cell cultures using the DPP-4 drug discovery Kit (Enzo Life Sciences, Farmingdale, NY, USA) with the Gly-Pro-para-nitroaniline (pNA) chromogenic substrate, according to the manufacturer’s instructions.

### Gene expression analysis

After 7 days of culture total RNA was extracted from PACs using RNeasy kit (Qiagen), following the manufacturer’s protocol. RNA quantity was determined on a Nanodrop Spectrometer (termo Fisher scientific Inc) (using 1 OD260 = 40 μg RNA). A260/A280 ratios were also calculated for each sample. RNA was reverse transcribed to generate cDNA using the First-Strand cDNA Synthesis Kit from Invitrogen following the manufacturer’s protocol. Samples were mixed by vortexing and briefly centrifuged and denaturated by incubation for 5 minutes at 70°C to prevent secondary structures of RNA. Samples were incubated on ice for 2 minutes to allow the primers to align. Gene-specific primer pairs were designed using Primer-BLAST (NCBI) and were each validated prior to use by gradient PCR and gel analysis to test for optimal annealing temperature, reaction efficiency and specificity (Table 1). Duplicates of sample cDNA were then amplified on the 7900HT Fast Real-Time PCR System (Applied Biosystems) using the Fast SYBR Green RT-PCR kit (Applied Biosystems) in 96-wells plates (micro amp optical, Applied Biosystems). Expression data were normalized to the mean of housekeeping gene to control the variability in expression levels and were analyzed using the 2^-ΔCT^ method.

**Table 1:** Primer sequences for real time PCR analysis

**Gene**	**FW primer sequence**	**RV primer sequence**
β-Actin	AGAGCTACGAGCTGCCTGAC	GGATGCCACAGGACTCCA
Bcl2	GTGGTGCAACCCACCACTTC	GGCAGGCATGTTGACTTCAC
CDKN1A	AGCTATGACCTCAAGGACAC	CGGCGTTTGGAGTGGTAGAA
CXCR4	GAAACCCTCAGCGTCTCAGT	AGTAGTGGGCTAAGGGCACA
IL8	TGTGAAGGTGCAGTTTTGCCA	CCCAGTTTTCCTTGGGGTCC
MCP1	ACAACACGCTGTTCGGCTA	GGGGCATTGATTGCATCTGG
DPP4	ACGTGAAGCAATGGAGGCAT	GTGACCATGTGACCCACTGT
MMP9	AGAGCTACGAGCTGCCTGAC	TGGGTGTAGAGTCTCTCGCT
TGF alpha	TGAAAGCATGATCCGGGACG	TGGGGAACTCTTCCCTCTGG
VCAM1	GTTTGCAGCTTCTCAAGCTTT	GATGTGGTCCCCTCATTCGT
ICAM1	TGTGACCAGCCCAAGTTGTT	TGGAGTCCAGTACACGGTGA
ITGB2	GTGGTGCAACCCACCACTTC	GCATGTCCCTCGGTGTGCT

### Mouse colony forming assay

Hematopoietic colonies were grown from unfractioned bone marrow cells of C57Bl/6 mice and quantified using the Methocult system (Stem Cells inc. Vancouver, Canada). For all experiments using animals, the ‘Principles of laboratory animal care’ (NIH publication no. 85–23, revised 1985; http://grants1.nih.gov/grants/olaw/references/phspol.htm) as well as specific national laws were followed.

### Spheroid assay

For preparation of methocoel, 6 g of methyl-cellulose together with a magnetic stir bar were autoclaved in a 500 ml flask. Afterwards, 250 ml of 60°C basal EBM medium was added under sterile conditions and the suspension was stirred at 60°C for 40 min. Additional 250 ml of basal EBM medium were added and the solution was stirred at 4°C overnight. 50 ml portion of the solution were centrifuged for 2 h at 4000 rpm at room temperature. The highly viscose soluble fraction was separated from insoluble residue and was stored at 4°C. The following protocol was applied for the spheroid assay: 48,000 HUVEC were mixed with 6 ml of methocoel/HUVEC medium and seeded as 100 μl drops in a 96-well U-bottom dish using a multipette. The cells were incubated for 24 h in an incubator to form spheroids. The day after, spheroids were collected, centrifuged and the spheroid pellet was mixed with methocoel-mix The collagen gel was prepared on ice and 500 μl of collagen gel were added to the spheroid/methocoel solution, mixed by pipetting and seeded on a 24-well culture dish for 30 min in an incubator. The spheroids were cultured for 24 h at 37°C and 5% CO_2_. Spheroids were fixed with formaldehyde. For quantification, 10 spheroids were assessed for cumulative sprout length, number of sprouts and number of branch points.

### In vivo angiogenesis assays

To gather information on the presence of functional circulating PACs and how they are modulated by Saxagliptin in type 2 diabetic subjects, we used the in vivo Matrigel plug angiogenesis assay with patients’ PBMC. Briefly, PBMC were isolated with Histopaque (Sigma-Aldrich). Cell count and viability were assayed with an automated BioRad TC20 cell counter. Then, 3 × 10^6^ PBMC were resuspended in 500 μL phenol-free Matrigel (BD, cat no. 356237) and implanted subcutaneously into the dorsum of immunodeficient RAG-2/gamma(c) double knock-out mice (in-house colony). Experiments involving animals were performed according to national guidelines and according to the ‘Principles of laboratory animal care’ (NIH publication no. 85–23, revised 1985; http://grants1.nih.gov/grants/olaw/references/phspol.htm). The experiment was performed with PBMC of n = 5 type 2 diabetic patients on Saxaglipthin therapy (>4 months) and n = 5 type 2 diabetic patients on non-incretinergic therapy. Mice were anesthetized with 100 mg/ml Ketamine HCL and 20 mg/ml Xylazine. To minimize variability, the same mouse received Matrigel plugs from a Saxagliptin-treated and a control patient. Plugs were explanted 10 days later for macroscopic inspection, histology (H&E staining), and determination of the hemoglobin/protein content ratio (Drabkin’s solution and Bredford reagent respectively, Sigma-Aldrich), which is as a surrogate of perfusion.

In addition to the traditional Matrigel plug assay, we also used the Directed In Vivo Angiogenesis Assay (DIVAA, Amsbio, Abingdon, UK), which employs semi-closed small silicone cylinders known as angioreactors, filled with Matrigel with or without a growth factor cocktail (fibroblast growth factor [FGF] + vascular endothelial growth factor [VEGF]) specifically design to stimulate vascular invasion. Angioreactors containing patients’ PBMC were implanted subcutaneously in the dorsal flanks of RAG-2/gamma(c) double knock-out mice. Compared to the traditional plug assay, the sleek design of angioreactors provides a standardized platform for reproducible and quantifiable in vivo angiogenesis assays and prevents assay errors due to absorption of Matrigel by the mouse. In the present protocol, each anaesthesized mouse received implantation of 4 angioreactors, 2 containing cells from Saxagliptin-treated patients and 2 containing cells from control patients, each with or without adding growth factors. Angioreactor tubes were explanted 10 days later for gross inspection of vascular invasion and determination of perfusion by FITC Lectin detection, according to the manufacturer’s protocol. As fluorescence labelled *Griffonia simplicifolia* Lectin I binds to alpha-D-galactosyl and N-acetyl galactosaminyl groups on the surface of endothelial cells, Lectin content is a measure of angioreactor tube vascular invasion.

### Statistical analysis

Data are presented as mean ± standard error, or as percentage where appropriate. Normal distribution of the variables under investigation was verified using the Kolmogorov-Smirnov test. Non normal variables were log transformed. Comparison between means was performed with the unpaired 2-tail Student’t *t* test. Comparison between more than 2 groups was performed with ANOVA. The Bonferroni correction was used to account for multiple testing, where appropriate. Frequencies were compared using the Chi square test. Statistical significance was accepted at p < 0.05.

## Results

### Characterization of cultured pro-angiogenic cells

**Figure 1:**
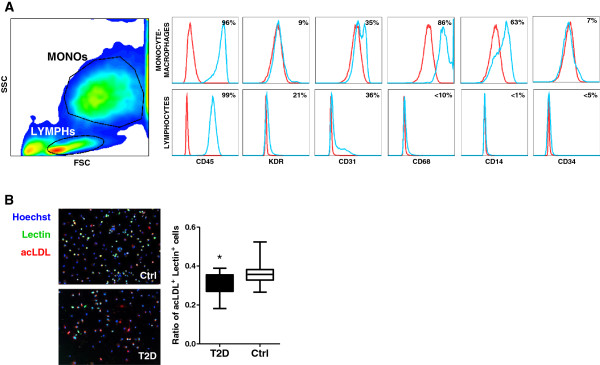
**Phenotypic characterization of cultured PACs. A)** PACs were characterized for surface marker expression by flow cytometry. In the side scatted (SSC) versus forward scatter (FSC) morphologic plot, lymphocytic cells (LYMPHs) and monocyte-macrophages (MONOs) were identified and gated separately. Histograms reporting the expression of relevant leukocyte (CD45, CD14, CD68) and endothelial markers (CD31, KDR, CD34) are shown, together with mean percent expression from 3 replicates. The red line indicates negative control, while the blue line indicates the stained condition. **B)** Cells in the PACs culture were stained with the endothelial markers acLDL and Ulex Lectin. The fraction of cells that were positive for both markers were compared in cultures obtained from T2D or healthy control cells. *p < 0.05 T2D vs Ctrl.

Pro-angiogenic cells (PACs) cultured from human PBMCs constitute a heterogenous population composed mostly of CD45^+^ lymphocytes and monocyte/macrophage lineage cells, plus a small population of stem/progenitor cells, possibly including EPCs [10]. Accordingly, we found that PACs culture was comprised of i) small cells in the lymphocytic morphologic (FSC vs SSC) gate, some of which expressed KDR (21 ± 3%) and CD31 (36 ± 5%) and can correspond to angiogenic T cells present in PACs/early EPCs culture [18]; ii) larger mononuclear cells expressing CD14, CD68, and CD31, likely belonging to the monocyte-macrophage population. The monocytic/lymphocytic cell ratio was 2.2 ± 0.1 and fairly constant throughout experiments. As expected, expression of the stem/progenitor cell marker CD34 was low (Figure 1A). These data indicate that cells used in subsequent experiments correspond to the PAC phenotype already described in the literature. Cultured PACs were also characterized by the double positivity for the (non-unequivocal) endothelial markers acetylated LDL uptake and Ulex-Lectin binding (Figure 1B). Interestingly, the fraction of acLDL^+^Lectin^+^ cells in the PACs culture was significantly lower in T2D compared to healthy controls (30.8 ± 0.8% vs 36.0 ± 0.1%; p = 0.003). There were no differences in the proportion of lymphocytic and monocytic cells in PACs cultures of T2D versus healthy subjects (not shown).

### In vitro DPP-4 activity sources and effective enzymatic inhibition

**Figure 2:**
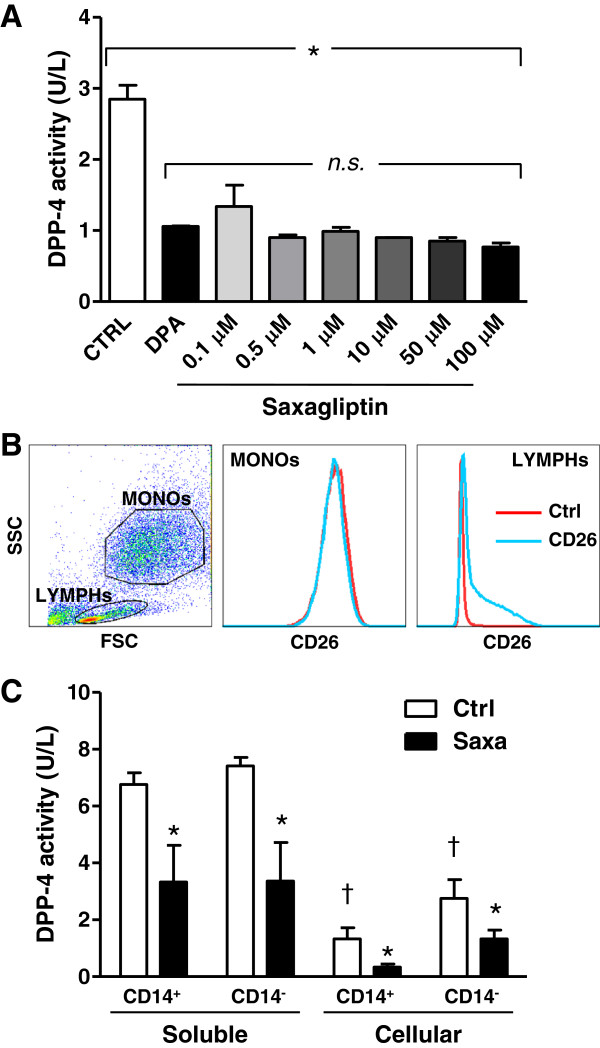
**In vitro DPP-4 inhibition. A)** Degrees of soluble DPP-4 inhibition in the conditioned medium by treatment of HUVECs with Diprotin-A (DPA) or different Saxagliptin concentrations, relative to the untreated control condition (CTRL). The experiment was performed in triplicate. *p < 0.05 for ANOVA test. **B)** Expression of membrane DPP-4 (CD26) on monocytic (MONOs) and lymphocytic (LYMPHs) cells, identified by the side scatted (SSC) versus forward scatter (FSC) morphologic plot of PACs culture. **C)** Soluble and cellular DPP-4 activity and the extent of its inhibition of Saxagliptin were tested in PACs cultured from CD14^pos^ and CD14^neg^ preselected cells. *p < 0.05 for CD14^-^ versus CD14^+^; †p < 0.05 for cellular versus soluble (n = 3 replicates).

We preliminarily tested the non-pharmacologic DPP-4 inhibitor Diprotin-A and several concentrations of the clinically available inhibitor Saxagliptin on DPP-4 activity in the conditioned medium obtained from HUVEC cultures. We found that 0.5 μM Saxagliptin was sufficient to reach maximal in vitro DPP-4 inhibition (~70%), which was equal to the degree of inhibition achieved using standard Diprotin-A concentrations (Figure 2A). The 0.5 μM Saxagliptin concentration, which has the same order of magnitude as the C_max_ obtained in vivo in humans after a 5 mg oral dose (0.1-0.3 μM), was chosen for subsequent experiments.

DPP-4 exists as a soluble secreted protein or a membrane-bound isoform, which also plays a role in signal transduction and adenosine metabolism [19]. In cultured PACs, membrane DPP-4 (CD26) expression was restricted to a subpopulation of lymphocytes (~50% of the lymphocyte gate), whereas larger monocytic cells were CD26-negative (Figure 2B). By using PACs cultured from CD14^pos^ or CD14^neg^ cells, we compared soluble (cell-free conditioned medium) and cellular (cell extracts) DPP-4 activity and the effect of Saxagliptin. Soluble DPP-4 activity was 3–5 fold higher compared to cellular DPP-4 activity regardless of whether CD14^pos^ or CD14^neg^ cells were present in the culture, and was inhibited >50% by Saxagliptin. As expected, cellular DPP-4 activity was higher in CD14^neg^(CD26^+^) cells compared to CD14^pos^(CD26^-^) cells and it was inhibited >50% by Saxagliptin. The DPP-4 activity of CD14^pos^ cells, which do not express significant amounts of membrane CD26, can be explained by residual DPP activity provided by isoforms other than DPP-4 (e.g. DPP-8 and -9) [20], that are partly inhibited by Saxagliptin (Figure 2C). These data indicate that, in PAC cultures, soluble activity is higher than cellular activity of DPP-4, the latter being mostly attributed to lymphocytes than to monocytic cells, while both soluble and cellular DPP-4 are equally inhibited by Saxagliptin.

### Functional effects of Saxagliptin on PACs from healthy controls and type 2 diabetic patients

We obtained PBMCs from n = 20 healthy blood donors (50% males, aged 52 ± 3) free of diabetes and cardiovascular disease, and n = 20 patients with T2D (Table 2). We tested in vitro the ability of Saxagliptin 0.5 μM to modify PACs properties, such as clonogenesis and proliferation, adhesion, migration and tubulization. Given that DPP-4 inhibition is supposed to affect PACs by protecting SDF-1α (and other substrates) from enzymatic inactivation [15], we evaluated the effects of Saxagliptin with and without SDF-1α supplementation.

**Table 2:** Characteristics of type 2 diabetic patients included in the study

**Variable**	**In vitro study**	**In vivo study**	
		**Saxagliptin**	**Controls**
Number	20	5	5
Age, years	65.2 ± 2.3	67.4 ± 2.6	66.1 ± 1.1
Sex male, %	65.0	80.0	60.0
Risk factors			
BMI, kg/m^2^	30.3 ± 2.1	29.1 ± 1.9	28.7 ± 2.1
Waist circumference, cm	104.8 ± 3.9	102.8 ± 4.0	103.7 ± 5.1
Diabetes duration, years	11.3 ± 4.2	9.8 ± 3.6	12.4 ± 5.3
Fasting glucose, mg/dl (mmol/l)	166.2 ± 8.1	141.2 ± 6.7	138.8 ± 4.5
	(9.2 ± 0.5)	(7.8 ± 0.4)	(7.7 ± 0.2)
HbA1c, % (mmol/mol)	8.4 ± 1.1	7.6 ± 1.6	7.8 ± 1.2
	(68.3 ± 9.0)	(59.6 ± 12.5)	(61.7 ± 9.5)
Hypertension, %	75.0	80.0	100.0
Systolic blood pressure, mm Hg	138.1 ± 3.2	137.4 ± 4.2	140.1 ± 3.5
Diastolic blood pressure, mm Hg	86.7 ± 2.7	82.4 ± 3.8	84.5 ± 4.6
Total cholesterol, mg/dl (mmol/l)	188.2 ± 5.1	172.5 ± 4.9	185.4 ± 6.7
	(4.9 ± 0.1)	(4.4 ± 0.1)	(4.8 ± 0.1)
HDL cholesterol, mg/dl (mmol/l)	46.6 ± 2.2	48.2 ± 1.1	46.9 ± 0.8
	(1.2 ± 0.1)	(1.2 ± 0.02)	(1.3 ± 0.02)
LDL cholesterol, mg/dl (mmol/l)	98.1 ± 4.9	81.2 ± 3.2	88.4 ± 2.9
	(2.5 ± 0.1)	(2.1 ± 0.1)	(2.7 ± 0.07)
Triglycerides, mg/dl (mmol/l)	149.3 ± 12.4	162.1 ± 14.8	138.2 ± 16.3
	(1.7 ± 0.1)	(1.8 ± 0.2)	(1.6 ± 0.2)
Smoke, %	15.0	0.0	20.0
Complications			
Coronary artery disease, %	25.0	20.0	0.0
Peripheral vascular disease, %	20.0	40.0	20.0
Cerebrovascular disease, %	40.0	40.0	20.0
Retinopathy, %	20.0	0.0	20.0
Nephropathy, %	25.0	0.0	0.0
eGFR < 60 ml/min/1.73 mq	0.0	0.0	0.0
Therapy			
Insulin therapy, %	50.0	0.0	20.0
Oral therapy, %	45.0	100.0	80.0
Diet alone, %	10.0	0.0	20.0
ACE inhibitors/ARBs, %	80.0	80.0	80.0
Statin, %	65.0	60.0	80.0

Data are presented as mean ± standard error, or as percentage, where appropriate. There were no significant differences between the two groups of the in vivo study.

#### Gene expression

**Figure 3:**
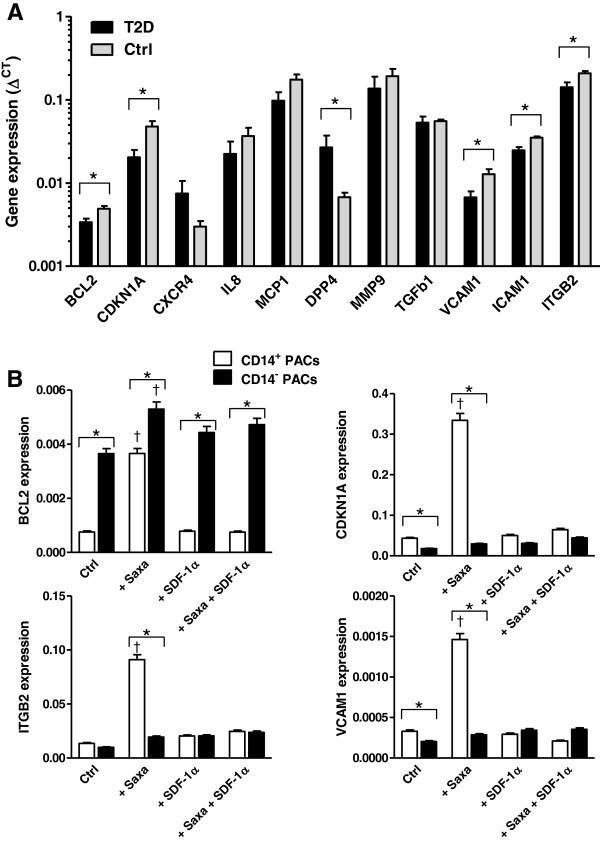
**Gene expression analysis of diabetic and healthy PACs. A)** Gene expression was determined in the whole PACs population obtained from type 2 diabetic patients (T2D) and healthy subjects (Ctrl). Expression is reported relative to the housekeeping gene as 2^ΔCT^. Note the logarithmic scale. *p < 0.05 T2D versus Ctrl. **B)** Expression of a selected number of genes found to be differentially expressed in (A) was tested in CD14^+^ monocytic and in CD14^-^ lymphocytic PACs separately. *p < 0.05 in CD14^+^ vs CD14^-^ cells; ^†^p < 0.05 versus the control condition.

First, we determined the expression of genes related to PACs function and survival. In PACs isolated from T2D patients compared to healthy control PACs, we found significantly altered expression of BCL2, CDKN1A, VCAM1, ICAM1, ITGB2 and DPP4 (Figure 3A). Importantly, as previously noted [21], DPP4 expression was markedly increased in diabetic PACs, providing a rationale for pharmacologic DPP-4 inhibition. Expression of the selected genes in the entire PACs culture was unaffected by treatment with Saxagliptin and/or SDF-1α (not shown). In separated CD14^+^ monocytic PACs compared to CD14^-^ lymphocytic PACs, we found lower expression of BCL2 and higher expression of CKDN1A and VCAM1 at baseline (Figure 3B). In addition, Saxagliptin treatment increased BCL2, CDKN1A, ITGB2 and VCAM1 expression in CD14^+^ PACs, while the effect was modest or absent in CD14^-^ PACs.

#### Proliferation and clonogenesis

**Figure 4:**
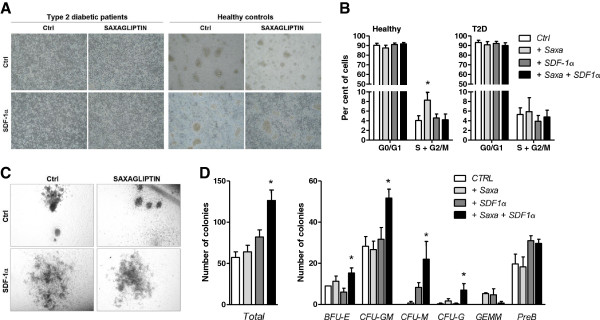
**Effects of Saxagliptin on clonogenesis and proliferation. A)** Representative microphotographs of PACs culture from type 2 diabetic patients and healthy controls exposed to Saxagliptin with or without SDF-1α supplementation. **B)** Analysis of cell cycle phases in cultured PACs of healthy subjects (left) and type 2 diabetic patients (right). *p < 0.05 versus the unstimulated control condition (Ctrl). **C)** Representative microphotographs of murine hematopoietic colonies exposed to Saxagliptin with or without SDF-1α supplementation. **D)** Quantification of total (left) and types (right) of hematopoietic colonies in relation to treatment with Saxagliptin, with or without SDF-1α supplementation. *p < 0.05 versus the unstimulated control condition (Ctrl).

While the culture of PACs from healthy controls yielded several rounded cell colonies in addition to interspersed cells, colonies were rarely seen during culture of PACs from T2D (Figure 4A). As expected from a PACs culture, the proliferation rate was low (3-5%). Treatment with Saxagliptin alone, but not in combination with SDF-1α, doubled the percentage of cells in the S + G2/M phases compared to the control condition only in healthy subjects’ PACs (Figure 4B). It has been previously reported that DPP-4 cleaves and inactivates several hematopoietic growth factors, suggesting that DPP-4 inhibition may sustain hematopoiesis [22]. Saxagliptin and/or SDF-1α did not restore colony formation during PAC culture from T2D PBMCs, but tended to increase the number of colonies of PACs cultured from healthy controls. Furthermore, using the methylcellulose mouse bone marrow hematopoietic colony assay (Figure 4C), we report that treatment with Saxagliptin + SDF-1α was indeed able to increase the total number of hematopoietic colonies. A breakdown analysis showed that treatment with Saxagliptin + SDF-1α induced consistent trend increases of most colony types, especially granulocytes and macrophages (Figure 4D). These data indicate that, unlike in diabetic cells, in non diabetic cells Saxagliptin may favour proliferation, while the co-treatment with SDF-1α promotes hematopoietic clonogenesis. Differences in the proliferative response between healthy control and diabetic PACs may be related to the differential baseline expression of genes regulating cell cycle and survival, such as CDKN1A (encoding p21) and BCL2 (Figure 3). Saxagliptin and/or SDF-1α did not significantly affect the percentage of acLDL^+^Lectin^+^ cells (not shown).

#### Adhesion

**Figure 5:**
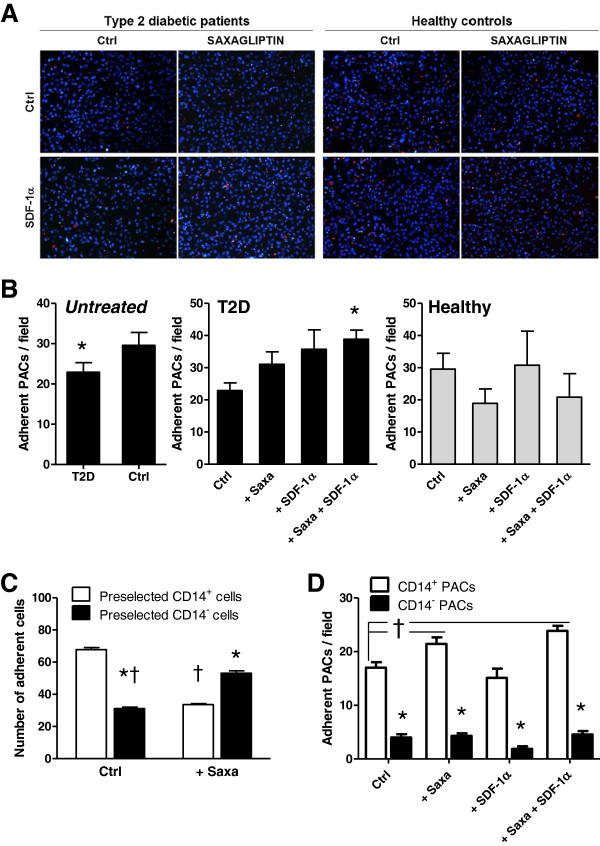
**Effects of Saxagliptin on adhesion of PACs. A)** Representative microphotographs of red-labelled PACs adhering onto HUVEC monolayers (nuclei labelled in bue with Hoechst), using cells from type 2 diabetic patients and healthy controls exposed to Saxagliptin with or without SDF-1α supplementation. **B)** Quantification of adherent PACs from type 2 diabetic (T2D) and healthy (Ctrl) subjects in the untreated condition and according to the treatment with Saxagliptin, with or without SDF-1α supplementation. *p < 0.05 versus the untreated control condition (Ctrl). **C)** Effects of Saxagliptin on cell adhesion was determined in cultures of healthy PACs obtained from pre-selected CD14^+^ and CD14^-^ cells. *p < 0.05 versus the untreated control condition (Ctrl). **D)** Baseline adhesion and effects of Saxagliptin +/- SDF-1α was tested in separated CD14^+^ and CD14^-^ PACs, after cells were cultured from unselected PBMCs. *p < 0.05 in CD14^+^ vs CD14^-^ cells; ^†^p < 0.05 versus the control condition.

Adhesion of PACs to mature endothelial cells represents an important step for the pro-angiogenic and vascular repairing activity of PACs. We thus tested adhesion of PACs to HUVECs *in vitro* and found that the number of adherent PACs was unaffected by treatment in healthy controls, while Saxagliptin significantly increased adhesion of T2D PACs in the presence of SDF-1α (Figure 5A, B). The significantly lower baseline adhesive capacity of diabetic PACs compare to control PACs and the differential response to Saxagliptin + SDF-1α may be related to the different baseline expression of adhesion molecule genes, such as ICAM1, VCAM1 and ITGB2 (Figure 3).

We used this assay to understand the relative contribution of membrane bound DPP-4 (CD26), which is expressed on CD14^neg^, but not on CD14^pos^ PACs. Therefore, we cultured PACs from healthy controls starting from immunomagnetically purified populations of CD14^pos^ and CD14^neg^ cells. We found that Saxagliptin reduced adhesion by CD14^pos^CD26^neg^ cells and increased adhesion by CD14^neg^CD26^pos^ cells (Figure 5C). This indicates that inhibition of soluble DPP-4 only (as in cultures of CD14^pos^ cells) and inhibition of both isoforms (as in cultures of CD14^neg^ cells) can have opposing effects on PACs function and explains, at least in part, why Saxagliptin has modest or no overall net effect on the heterogeneous PACs culture. In addition, to better understand which is the saxagliptin-responsive cell type in the heterogeneous PACs culture, we separated CD14^+^ from CD14^-^ PACs cultured from unselected PBMCs in the presence of Saxagliptin and/or SDF-1α. We found that the adhesive capacity of CD14^+^ monocytic PACs was significantly higher than that of CD14^-^ lymphocytic cells, and that it was stimulated by Saxagliptin with or without SDF-1α (Figure 5D).

#### Migration

**Figure 6:**
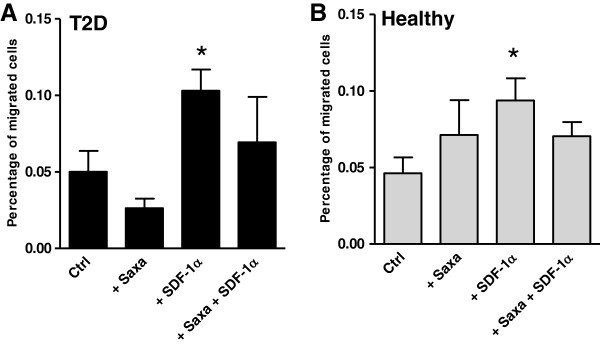
**Migration of PACs cultured from type 2 diabetic patients (A) and healthy controls (B) in the presence and in the absence of Saxagliptin and SDF-1α was assessed using a transwell system.** *p < 0.05 versus the untreated control condition (Ctrl).

PACs migration was assessed using a transwell assay, with the chemokine SDF-1α being the prototypical positive control [13]. We found that the percentage of migration was increased by SDF-1α using both healthy and diabetic PACs, whereas Saxagliptin alone or in combination with SDF-1α did not affect migration of both control and diabetic PACs (Figure 6). It should be noted that the absolute numbers of migrating cells in this system was low, thus limiting the chance of reporting small effects as significant.

#### Tube formation

**Figure 7:**
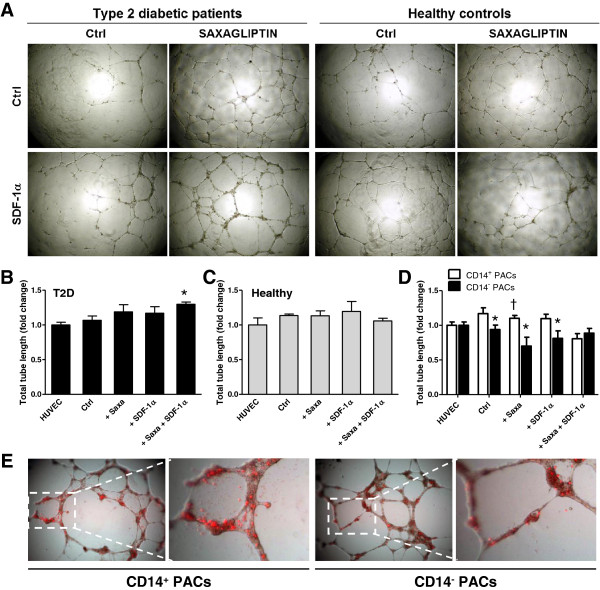
**Effects of Saxagliptin on migration and tubulisation of PACs. A)** Representative microphotographs of tube formation in 2D Matrigel by HUVECs co-cultured with PACs from type 2 diabetic patients and healthy controls exposed to Saxagliptin with or without SDF-1α supplementation. **B-C)** Quantification of total tube length formation by HUVECs alone and HUVECs co-incubated with PACs either untreated (Ctrl) or treated with Saxagliptin and/or SDF-1α. Panel B shows data obtained with PACs from type 2 diabetic patients, while panel C shows data obtained with healthy PACs. *p < 0.05 versus HUVECs alone, set at 1.0. **D)** Quantification of total tube length formation by HUVECs alone and HUVECs co-incubated with CD14^+^ or CD14^-^ PACs from healthy controls. *p < 0.05 in CD14^+^ vs CD14^-^ cells; ^†^p < 0.05 versus the control condition. **E)** Red-labelled PACs co-cultured with HUVECs in the tube forming assay were imaged under an inverted fluorescence micriscioe to detect their spatial localization and quantify tubes with integrated CD14^+^ and CD14^-^ PACs.

Tubulization by HUVECs co-cultured with PACs was assessed using the 2D Matrigel assay (Figure 7A). It is well known that PACs do not spontaneously form tubules in 2D Matrigel, but can sustain tubulogenesis by mature endothelial cells [10]. We found that only T2D PACs treated with Saxagliptin + SDF-1α were able to increase tube length formation by HUVECs, while there was no effect on healthy PACs (Figure 7B-C). The number of branching points was not significantly different in T2D compared to controls and was unaffected by treatments (not shown). In addition, we evaluated the tube supportive capacity of CD14^+^ PACs versus CD14^-^ PACs and how they are affected by treatments. We found that HUVECs co-cultured with CD14^+^ monocytic PACs showed increased tube formation compared to co-culture with CD14^-^ lymphocytic PACs. Moreover, tube formation by HUVECs/CD14^+^ PACs was significantly increased by Saxagliptin treatment compared to the control condition (Figure 7D). Most of labelled PACs co-cultured with HUVECs remained located in cellular islands at tube intersections, while only some contributed physically to tube formation. No difference was seen in the percentages of tubes bearing integrated CD14^+^ (15 ± 4%) versus CD14^-^ PACs (12 ± 2%; p = 0.41) (Figure 7E).

**Figure 8:**
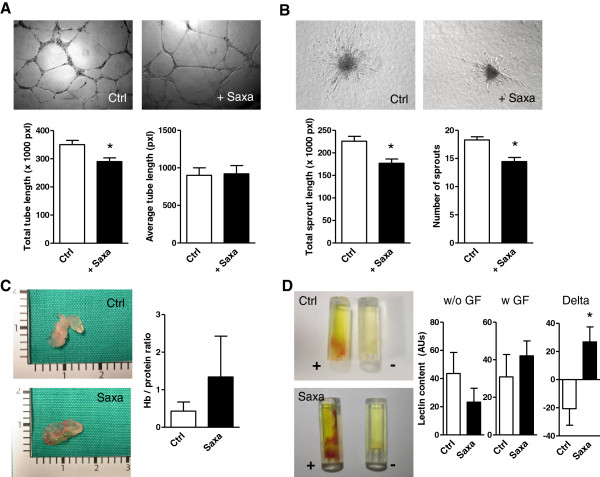
**Effects of Saxagliptin on angiogenesis. A)** The effect of Saxagliptin on tube formation by HUVECs in 2D Matrigel was assessed. Total tube length as well as average tube length are reported. *p < 0.05 (n = 3 replicates) for Saxagliptin-treated versus the untreated control condition (Ctrl). **B)** The effect of Saxagliptin on sprouts formation by spheroids of HUVECs was assessed. Total sprouts length as well as the number of sprouts are reported. *p < 0.05 (n = 3 replicates) for Saxagliptin-treated versus the untreated control condition (Ctrl). **C)***In vivo* angiogenesis in Matrigel plugs by PBMCs isolated from Saxagliptin-treated and non-incretinergic drug treated type 2 diabetic patients (n = 5/group). Representative figures, as well as quantification of Hb content are shown. **D)** PBMC from Saxagliptin-treated and non-incretinergic drug treated type 2 diabetic patients (n = 5/group) were also used for the Directed In Vivo Angiogenesis Assay (DIVAA) using angioreactor tubes with (+) and without (-) growth factors (GF). After visual inspection, Lectin content was determined as an indicator of vascular invasion of the tube. Lectin content quantification in tubes without and with GF, as well as the difference between the two are shown. *p < 0.05 Saxa versus Ctrl.

Finally, as the tube assay is strictly dependent on the cooperation between HUVECs and PACs, we explored the effect of Saxagliptin on *in vitro* angiogenesis assays of isolated HUVECs. We found that, using both the 2D Matrigel tubulisation assay and the spheroid sprouting assay, Saxagliptin modestly but significantly reduced *in vitro* angiogenesis by mature endothelial cells (Figure 8A, B).

### In vivo effect of Saxagliptin on angiogenesis induced by circulating cells

To assess the relevance of the pro-angiogenic effects of Saxagliptin on circulating PACs compared to the possible anti-angiogenic effects on mature ECs, we used *in vivo* Matrigel assays. First, PBMCs from T2D treated with Saxagliptin (n = 5) and PBMCs from T2D patients on non-incretinergic therapy (n = 5; see Table 2) were embedded into Matrigel plugs and implanted subcutaneously into immunodeficient mice. Angiogenesis in this model is mediated by the cooperation between the intraplug cells (the circulating source of PACs) and mouse ECs invading the plug. We found a non-significant increase in vascular plug invasion and hemoglobin content (a surrogate of plug vascularization) in plugs containing PBMC from Saxagliptin-treated compared to those from untreated diabetic patients (Figure 8C). To further address this issue, we used the Directed In Vivo Angiogenesis Assay (DIVAA), which allows a more reproducible quantification of *in vivo* angiogenesis. DIVAA angioreactor tubes were implanted with cells obtained from saxagliptin-treated and non incretin drug-treated T2D patients, with or without growth factors (GF) that stimulate angiogenesis. We did not find significant differences in the Lectin content (a proxy of perfusion in DIVAA experiments) in tubes containing cells from saxagliptin-treated patients versus controls in either the presence or absence of GF. However, the difference between Lectin content in tubes with and without GF, indicative of the ability of GF to increase PBMC-mediated angiogenesis in each patient, was significantly higher (p = 0.03) for saxagliptin-treated patients compared to controls (Figure 8D).

## Discussion

In the present study, we show that DPP-4 inhibition with Saxagliptin reverses PACs dysfunction associated with T2D *in vitro* and improves inducible angiogenesis by patients’ cells *in vivo*. We have previously shown that DPP-4i increases circulating EPCs in a similar population of T2D subjects [15]. Herein, we add to those findings showing that, not only the level, but also the function of vascular protective cells can be improved by DPP-4i treatment.

PACs derived from PBMC cultures differ from EPCs quantified *ex vivo* by flow cytometry, as they are composed of a heterogenous population of angiogenic T cells and monocyte-macrophages, plus a small population of progenitor cells [10]. The pro-angiogenic and vascular repair capacity of human PACs has been consistently demonstrated in pre-clinical studies [23,24]. In addition, autologous administration of bone marrow derived PACs was shown to improve left ventricular ejection fraction in patients with acute myocardial infarction [25], confirming the protective cardiovascular effects of these cells in humans. The mechanisms whereby PACs achieve vascular protection *in vivo* are not entirely clear, but data suggest that they are mainly derived from an intense paracrine activity, rather than definite endothelial differentiation and integration [2]. Indeed, although a significant ontologic overlap between the endothelium and hematopoietic cells exists in the embryo [26], epigenetic brakes prevent blood-derived PACs to differentiate into mature endothelium in adulthood [27]. The heterogeneous composition of PACs culture could be seen as a limit to the interpretation of the present findings. However, it should be noted that late EPCs and ECFC can be only stochastically isolated from peripheral blood of diseased subjects [10], thus limiting reproducibility of the findings. Whether PACs mainly represent an *in vitro* artefact or they also exist *in vivo* is a matter of debate, although the discovery of the so-called haemogenic endothelium suggest that endothelial-hematopoietic overlaps can occur also in adulthood [26].

Several authors have reported that diabetes induces PACs dysfunction, through epigenetic changes [28], eNOS modulation [29], and humoral factors [30]. We herein confirm that PACs isolated from T2D have impaired differentiation, clonogenesis and adhesion compared to PACs isolated from healthy controls. This was associated with changes in the expression of genes related to adhesion and regulation of cell cycle. Such differences, however, cannot be directly attributed to diabetes per se, because T2D patients were also older than controls and had additional cardiovascular risk factors. The rationale for including healthy controls instead of matched non-diabetic patients was to assess the effects of Saxagliptin within each group and understand whether DPP-4i influences both diseased and healthy PACs. Interestingly, we found that, with the exception of proliferation, only PACs from T2D patients improved their function after treatment with Saxagliptin. This can be attributed to the marked upregulation of DPP-4 gene expression in diabetic PACs. We have previously shown that DPP-4 activity is increased in serum/plasma of T2D compared to non-diabetic patients and is not directly related to glucose control [21]. DPP-4 exists as either a soluble or membrane bound (cellular, CD26) isoform and the relative contribution of the 2 to the total DPP-4 activity and its biological effects were previously unknown. We show that soluble DPP-4 activity is higher than cellular DPP-4 activity, which is restricted to a lymphocyte subpopulation. This is particularly important in cultures of PACs, which are composed of angiogenic T cells and endothelial-like monocyte-macrophages. Indeed, we found that, though Saxagliptin did not significantly affect adhesion of healthy PACs, when PACs were isolated starting from CD14^+^(CD26/DPP-4^-^) cells or CD14^-^(CD26/DPP-4^+^) cells, the effects of Saxagliptin on adhesion were opposite. In addition, among the heterogeneous PACs population, CD14^+^ monocytic PACs compared to CD14^-^ lymphocytic PACs showed higher functionality at baseline and were much more responsive to Saxagliptin-induced gene expression changes and stimulation of adhesion and tube supporting capacity. While such differences can be the result of cell-type specific responses to Saxagliptin, it is possible that cellular DPP-4 inhibition exerts different effects compared to soluble DPP-4 inhibition. While membrane bound DPP-4 may transduce intracellular signals and is a cofactor of adenosine deaminase [19], secreted DPP-4 is supposed to act mainly through cleavage of soluble mediators. Indeed, we found that Saxagliptin improved whole PACs function only in the presence of SDF-1α supplementation, whereas Saxagliptin alone or SDF-1α alone less effective. Based on the low SDF-1α concentrations in the medium (fg/ml) compared to the high DPP-4 expression/activity, it is rationale that only simultaneous SDF-1α supplementation and DPP-4 inhibition provided significant biological effects, a finding that supports the mechanistic theory whereby DPP-4i affects PACs by protecting SDF-1α (and possibly other factors) from enzymatic degradation. In addition to improving PACs function, DPP-4 inhibition also mobilizes EPCs via SDF-1α [15]. This synergistic effect is expected to promote favourable outcomes in several diabetic complications [31], including wound healing [32,33].

In contrast to the positive effects exerted by Saxagliptin + SDF-1α on T2D PACs, Saxagliptin +/- SDF-1α did not affect function of healthy control PACs when evaluated in the entire population, but differentially affected function of CD14^+^ monocytic versus CD14^-^ lymphocytic cells. Saxagliptin also reduced angiogenesis by mature endothelial cells *in vitro*, suggesting cell-type specific effects. Therefore, to understand the overall net effects of Saxagliptin on angiogenesis *in vivo*, we used PBMCs isolated from Saxagliptin-treated and from control T2D patients treated with non-incretinergic drug. The Matrigel plug assay showed non-significantly higher perfusion obtained with Saxagliptin-treated compared to control cells. As this assay has wide variability depending on how the plug develops and is adsorbed in the mouse, we also used the more reliable and quantitative Directed In Vivo Angiogenesis Assay (DIVAA). We found that the extent to which growth factors (VEGF + FGF) increased vascular invasion of the angioreactor was significantly higher for Saxagliptin-treated compared to control cells. This suggests that Saxagliptin increases the ability of circulating cells to respond to pro-angiogenic growth factors, possibly protecting them from enzymatic degradation.

## Conclusions

Reversal of T2D PACs dysfunction and stimulation of inducible angiogenesis may translate in a microvascular [34] and cardiovascular protective activity of DPP-4i. While pooled data from short-term phase III randomized clinical trials in selected T2D patients showed potential cardiovascular benefit of Saxagliptin [35], the Saxagliptin Assessment of Vascular Outcomes Recorded in Patients with Diabetes Mellitus (SAVOR) clinical trial, conducted on >16,000 T2D patients with a history or risk factors for cardiovascular events, showed a neutral effect of Saxagliptin on the rate of ischemic events [36]. However, this event-driven study was terminated after just a median follow-up of 2.1 years, thus limiting the chance that protective effects of Saxagliptin translated into an event rate reduction. So far, experimental pre-clinical and clinical findings widely argue for potential cardiovascular protection by DPP-4i. The observation that Saxagliptin restores the function of PACs from T2D patients represents an additional step toward a better understanding of the pathobiology of DPP-4 in diabetes. PACs function can be restored by glucose normalization, as shown in islet-transplated type 1 diabetic patients [37]. Therefore, Saxagliptin may also impact PACs pro-angiogenic activity by improving glucose control [38]. As HbA1c was only slightly and not significantly lower in Saxagliptin-treated patients compared to patients on non-incretinergic therapies, pleiotropic extraglycemic effects are likely implicated.

## Abbreviations

APC: Allophycocyanin; CD: Cluster of differentiation; CMTMR: 5-(and-6)-(((4-chloromethyl)Benzoyl)Amino)tetramethylrhodamine; DIVAA: Directed in vivo angiogenesis assay; DPP: Dipeptidyl peptidase; EBM: Endothelial basal medium; EGM: Endothelial growth medium; eNOS: Endothelial nitric oxide synthase; EPCs: Endothelial progenitor cells; FGF: Fibroblast growth factor; FSC: Forward scatter; GF: Growth factors; GIP: Glucose-dependent insulinotropic peptide; GLP: Glucagon-like peptide; HUVECs: Human umbilical vein endothelial cells; MACS: Magnetic activated cell sorting; PACs: Proangiogenic cells; PBMC: Peripheral blood mononuclear cells; PE: Phycoerythrin; SAVOR: Saxagliptin assessment of vascular outcomes recorded in patients with diabetes mellitus; SDF: Stromal cell derived factor; SSC: Side scatter; T2D: Type 2 diabetes; VEGF: Vascular endothelial growth factor.

## Competing interests

GPF and AA received lecture or consultancy fees from companies with commercial interests in DPP-4 inhibitors, including the Saxagliptin manufacturer Bristol-Meyer-Squibb.

## Authors’ contributions

NP, MA, LM and RC conducted experiments, researched and interpreted data from in vitro and in vivo experiments. AA provided support, supervised the project and drafted the manuscript. GPF provided support, designed experiments, analyzed data and wrote the manuscript. All authors read and approved the final manuscript.
